# Supplementary material for: Bayesian refinement of protein structures and ensembles against SAXS data using molecular dynamics
Source: PLoS Comput Biol. 2017 Oct 18;13(10):e1005800. doi: 10.1371/journal.pcbi.1005800 (PMC5662244; doi:10.1371/journal.pcbi.1005800)
Supplement: S1 Archive — (BZ2) [file pcbi.1005800.s011.tar.bz2 › gromacs_4.62_bayesian_saxs_refi/share/html/online/g_msd.html]

g\_msd


| g\_msd Main Table of Contents |

**VERSION 4.6  
Sat 19 Jan 2013**

---

### Description

g\_msd computes the mean square displacement (MSD) of atoms from
a set of initial positions. This provides an easy way to compute
the diffusion constant using the Einstein relation.
The time between the reference points for the MSD calculation
is set with -trestart.
The diffusion constant is calculated by least squares fitting a
straight line (D\*t + c) through the MSD(t) from -beginfit to
-endfit (note that t is time from the reference positions,
not simulation time). An error estimate given, which is the difference
of the diffusion coefficients obtained from fits over the two halves
of the fit interval.

There are three, mutually exclusive, options to determine different
types of mean square displacement: -type, -lateral
and -ten. Option -ten writes the full MSD tensor for
each group, the order in the output is: trace xx yy zz yx zx zy.

If -mol is set, g\_msd plots the MSD for individual molecules
(including making molecules whole across periodic boundaries):
for each individual molecule a diffusion constant is computed for
its center of mass. The chosen index group will be split into
molecules.

The default way to calculate a MSD is by using mass-weighted averages.
This can be turned off with -nomw.

With the option -rmcomm, the center of mass motion of a
specific group can be removed. For trajectories produced with
GROMACS this is usually not necessary,
as mdrun usually already removes the center of mass motion.
When you use this option be sure that the whole system is stored
in the trajectory file.

The diffusion coefficient is determined by linear regression of the MSD,
where, unlike for the normal output of D, the times are weighted
according to the number of reference points, i.e. short times have
a higher weight. Also when -beginfit=-1,fitting starts at 10%
and when -endfit=-1, fitting goes to 90%.
Using this option one also gets an accurate error estimate
based on the statistics between individual molecules.
Note that this diffusion coefficient and error estimate are only
accurate when the MSD is completely linear between
-beginfit and -endfit.

Option -pdb writes a .pdb file with the coordinates of the frame
at time -tpdb with in the B-factor field the square root of
the diffusion coefficient of the molecule.
This option implies option -mol.

### Files

| option | filename | type | description |
| --- | --- | --- | --- |
| **-f** | traj.xtc | Input | Trajectory: xtc trr trj gro g96 pdb cpt |
| **-s** | topol.tpr | Input | Structure+mass(db): tpr tpb tpa gro g96 pdb |
| **-n** | index.ndx | Input, Opt. | Index file |
| **-o** | msd.xvg | Output | xvgr/xmgr file |
| **-mol** | diff\_mol.xvg | Output, Opt. | xvgr/xmgr file |
| **-pdb** | diff\_mol.pdb | Output, Opt. | Protein data bank file |

### Other options

| option | type | default | description |
| --- | --- | --- | --- |
| **-[no]h** | bool | no | Print help info and quit || **-[no]version** | bool | no | Print version info and quit || **-nice** | int | 19 | Set the nicelevel || **-b** | time | 0 | First frame (ps) to read from trajectory || **-e** | time | 0 | Last frame (ps) to read from trajectory || **-tu** | enum | ps | Time unit: fs, ps, ns, us, ms or s || **-[no]w** | bool | no | View output .xvg, .xpm, .eps and .pdb files || **-xvg** | enum | xmgrace | xvg plot formatting: xmgrace, xmgr or none || **-type** | enum | no | Compute diffusion coefficient in one direction: no, x, y or z || **-lateral** | enum | no | Calculate the lateral diffusion in a plane perpendicular to: no, x, y or z || **-[no]ten** | bool | no | Calculate the full tensor || **-ngroup** | int | 1 | Number of groups to calculate MSD for || **-[no]mw** | bool | yes | Mass weighted MSD || **-[no]rmcomm** | bool | no | Remove center of mass motion || **-tpdb** | time | 0 | The frame to use for option -pdb (ps) || **-trestart** | time | 10 | Time between restarting points in trajectory (ps) || **-beginfit** | time | -1 | Start time for fitting the MSD (ps), -1 is 10% || **-endfit** | time | -1 | End time for fitting the MSD (ps), -1 is 90% |

---

http://www.gromacs.org  
gromacs@gromacs.org
